# High Temperature Stable Separator for Lithium Batteries Based on SiO_2_ and Hydroxypropyl Guar Gum

**DOI:** 10.3390/membranes5040632

**Published:** 2015-10-23

**Authors:** Diogo Vieira Carvalho, Nicholas Loeffler, Guk-Tae Kim, Stefano Passerini

**Affiliations:** 1 Helmholtz Institute Ulm (HIU), Helmholtzstrasse 11, 89081 Ulm, Germany; E-Mails: diogo.carvalho@kit.edu (D.V.C.); nicholas.loeffler@kit.edu (N.L.); 2 Karlsruhe Institute of Technology (KIT), P. O. Box 3640, 76021 Karlsruhe, Germany

**Keywords:** separator, hydroxypropyl guar gum (HPG), silicon dioxide (SiO_2_), lithium batteries, aqueous processing, thermal stability

## Abstract

A novel membrane based on silicon dioxide (SiO_2_) and hydroxypropyl guar gum (HPG) as binder is presented and tested as a separator for lithium-ion batteries. The separator is made with renewable and low cost materials and an environmentally friendly manufacturing processing using only water as solvent. The separator offers superior wettability and high electrolyte uptake due to the optimized porosity and the good affinity of SiO_2_ and guar gum microstructure towards organic liquid electrolytes. Additionally, the separator shows high thermal stability and no dimensional-shrinkage at high temperatures due to the use of the ceramic filler and the thermally stable natural polymer. The electrochemical tests show the good electrochemical stability of the separator in a wide range of potential, as well as its outstanding cycle performance.

## 1. Introduction

Since their first commercialization by Sony in 1991, lithium-ion batteries have played an increasingly important role as power sources for portable electronic devices, such as electric tools and personal computers, due to their high working voltage and energy density, long-term cycle life, and high efficiency [1,2]. Nowadays, lithium-ion batteries are also considered as alternative energy storage systems and power supplies for electrical vehicles (EVs), as well as hybrid vehicles (HEVs), paving the way for more sustainable transport. Several other applications of lithium-ion batteries are already in place worldwide [3]. Nonetheless, researchers are placing many efforts to improve several issues of this battery technology, among which safety and reduced cost are, for obvious reasons, the most important [4].

Focusing on the safety aspect, the separator plays a prominent role in lithium-ion batteries. Separators are porous membranes placed between the cathode and anode. These membranes have the function of preventing any physical contact between the electrodes and enable a facile mobility of lithium ions by hosting large amounts of ion-conductive liquid electrolyte. Any contact between the electrodes, namely, a short circuit, can lead to a thermal runaway and, in the worst case, fire and explosion might occur as a result of the increasing temperature inside the lithium-ion cell [5]. Short circuits can occur due to several reasons, such as lithium dendrite growth [6] and dimensional shrinkage of the separator under an unusual heating of the cell. Therefore, separators are required to maintain their physical and electrochemical properties, even at relatively high temperatures [7].

Nowadays, most of the commercially used separators for Li-ion batteries are made of polypropylene (PP) and polyethylene (PE) or combinations thereof. These membranes offer excellent mechanical strength and chemical stability, however, the low melting point of PP and, especially, PE leads to a poor thermal stability and costly cell assembly. In fact, typically, electrodes and separators have to be dried separately (and at different temperatures) prior to the lithium-ion battery assembly since PP/PE separators do not stand high drying temperatures without shrinkage. Hence, after drying electrodes and separators separately, Li-ion batteries are assembled under a controlled atmosphere, increasing the cost and the time for the cell assembly. Another issue of using PP/PE based separators is the poor affinity to Li-ion electrolytes, which leads to long wetting times in controlled atmosphere and, as a consequence, an increase of the battery’s final cost [8].

Ceramic-based membranes have been developed in order to improve the performance of PP/PE based separators. These membranes employ inorganic materials, such as SiO_2_, Al_2_O_3_, CaCO_3_, or their mixtures as a coating layer or filler [2,7,8,9,10,11]. They show good affinity with non-aqueous liquid electrolytes and very low, if any, dimensional shrinking due to the high thermal stability of the employed particles. Moreover, these particles possess high thermal conductivity and, thus, dissipate heat quickly throughout the separator membrane [9]. Another advantage of using inorganic filler materials is a noticeable improvement in cycling performance of Li-ion cells due to their scavenging reaction with hydrofluoric acid (HF) traces, which stem from the reaction of the lithium salt (LiPF_6_) with residual moisture [12,13].

In this report, we propose a simple method for preparing a high temperature stable separator using silicon dioxide SiO_2_ particles and a low cost water-soluble polymeric binder. Due to the high abundance of SiO_2_ in the Earth’s crust, it is a low cost and environmentally friendly compound. This material is widely used in the electronic industry and is currently under development as an anode material for Li-ion batteries [14,15].

The binder used is this work is the non-ionic hydroxypropyl derivative of guar gum (HPG). Guar gums have been proved viable for electrochemical energy storage applications, such as binders for lithium titanium oxide (Li_4_Ti_5_O_12_ or LTO) [16] and silicon [17,18,19] anodes in lithium-ion batteries and gel polymer electrolytes in super capacitors [20]. The advantages of using HPG compared with native guar gum are the enhanced water solubility and thermal stability [21]. Additionally, HPG is a benign material, mostly used as thickening agent for cosmetics, food processing, the oil industry, and paper processing [22].

An environmentally friendly processing is described herein to prepare the high temperature stable membranes, which are then evaluated as separator for lithium-ion batteries. The membranes were characterized with respect to porosity, electrolyte wettability, electrochemical and thermal stabilities, and, finally, tested as separator in half-cells (Li metal negative electrode) with lithium nickel-manganese-cobalt oxide (LiNi_1/3_Mn_1/3_Co_1/3_O_2_ or NMC) and lithium titanate oxide (Li_4_Ti_5_O_12_ or LTO) based positive electrodes, since those two materials are already used in commercial lithium-ion batteries [3,16].

## 2. Results and Discussion

### 2.1. Membrane Characterization

After an extended exploration of compositions, self-standing homogeneous membranes were successfully prepared with the 80:20 weight ratio of SiO_2_ and HPG. Lower SiO_2_ contents (SiO_2_:HPG ≤ 70:30) led to non-porous membranes while lower HPG contents (SiO_2_:HPG ≥ 90:10) led to fragile and/or powdery samples.

**Figure 1:**
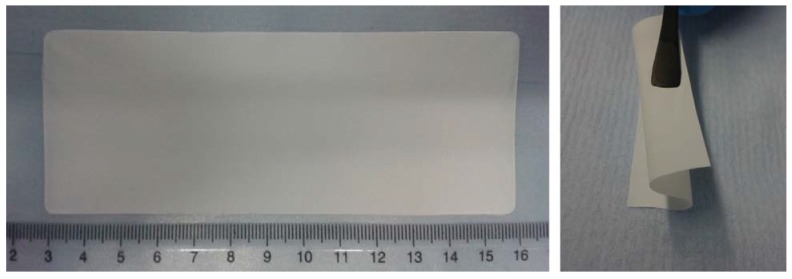
Images of the self-standing SiO_2_–HPG membrane after drying and removal from the PTFE tape. The good flexibility and self-integrity of the membrane is well evidenced in the right image.

Figure 1 depicts the SiO_2_–HPG membrane after drying at 20 °C (see Experimental Section for further details). The final membranes’ thickness ranged between 30 and 50 µm. The membranes appear quite homogeneous and uniform both in the bulk and the surfaces, *i.e.*, without binder or filler agglomerations when using highly viscous slurries (*i.e.*, having a gel appearance; HPG:H_2_O wt. ratio > 0.02), although the drying process takes much longer than with diluted (*i.e.*, very liquid-like; HPG:H_2_O wt. ratio < 0.01) slurries. This is because in the gelled slurries the drying process involve water diffusion through the membrane rather then binder segregation at the coating’s surfaces and/or filler sedimentation [23].

The homogeneity and uniformity of the membranes may also result from specific HPG–SiO_2_ interactions. In fact, HPG is formed by a carbon backbone with numerous functional hydroxyl groups. These may interact with sites forming on the surface of the SiO_2_ particles when dispersed in water. The active bonding, which mechanism (see below) was demostrated with FT-IR studies [17], probably starts during the slurry-mixing step but takes place mostly during the drying step (needed to remove all residual water [24]) as it is thermally activated.

$$\text{R}—\mathrm{OH}+\mathrm{Si}—\mathrm{OH}\overset{\Delta \text{t}}{\to }\text{R}—\text{O}―\mathrm{Si}+{\text{H}}_{2}\text{O}$$

The bonding improves the physical properties of the membrane also granting a good flexibility of the membrane (see Figure 1) and supporting the homogenous distribution of particles and binder.

The membrane’s porosity evaluated via the hexadecane infiltration method (see Experimental Section) was found to be about 52%, which is an appropriate value for separators in lithium-ion batteries [5,7].

**Figure 2:**
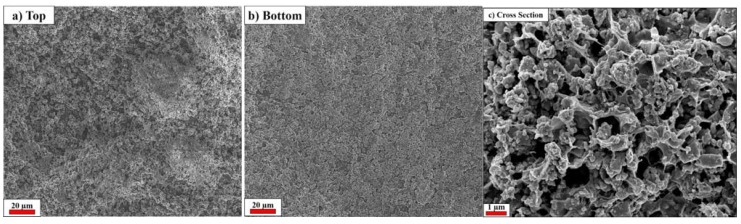
SEM pictures of the SiO_2_–HPG membrane. (**a**) Top face (air contact at drying step); (**b**) Bottom face (PTFE contact at drying step); (**c**) Cross section.

Figure 2 shows the SEM images of the membrane’s top and bottom surfaces as well as its cross section. The membrane shows very homogeneous top (air contact at drying step) (Figure 2a) and bottom (PTFE contact at drying step) (Figure 2b) surfaces, which appear suitable for lithium-ion battery application due to their quite uniform porosity distribution. In fact, non-homogeneous porosity leads to preferential redox reactions at specific areas on the electrodes’ surfaces, which may cause local heat evolution and/or lithium dendritic growth, resulting, e.g., in a cell short circuit [8]. As displayed in Figure 2a,b, the membrane possess only small pores <1 µm that can avoid the migration of particles, such as the active material particles, between the electrodes [5,7,9]. Figure 2c shows the SEM cross-sectional image of the membrane, showing as it is formed by a highly porous matrix with pores larger than 1 µm. Once impregnated by the electrolyte, the voluminous pores function as an electrolyte reservoir, improving the lithium ion path through the separator.

**Figure 3:**
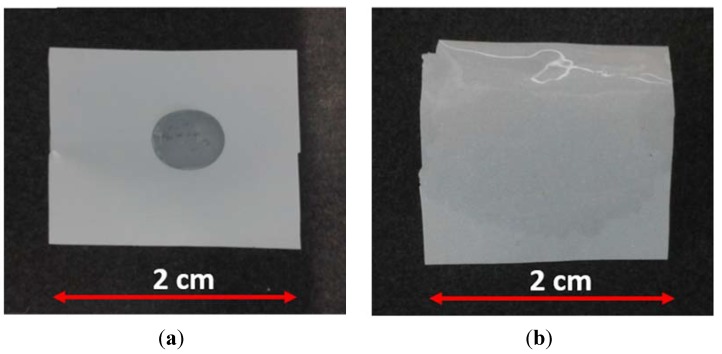
Images of membranes subjected to the wettability test. The pictures were taken 2 s after dropping 80 µL of electrolyte on the (**a**) commercial polyethylene (PE) single layer separator and (**b**) SiO_2_–HPG membrane.

Figure 3 depicts a typical test to investigate the wetting ability of separators. The commercial Polyethylene (PE) single layer separator were used as reference. A small amount of electrolyte (80 µL) was dropped on the membranes surface, which for the PE separator is possible to see a drop of the electrolyte (Figure 3a), while the SiO_2_–HPG was almost fully wetted after 2 s, as can be seen in Figure 3b. This is due to the good affinity of the SiO_2_ particles to organic-based electrolyte (1 M LiPF_6_ in EC:DMC = 1:1 *w*/*w*) [10]. The combination of excellent wettability, due to the hydrophilic characteristic of SiO_2_ particles [7,8,9,25] and the chemical structure of guar gum [16], and relatively high internal porosity resulted in the high absorbed volume of electrolyte in the separator (after 30 and 60 min the weight of the membrane increased by 290% and 370%, respectively).

**Figure 4:**
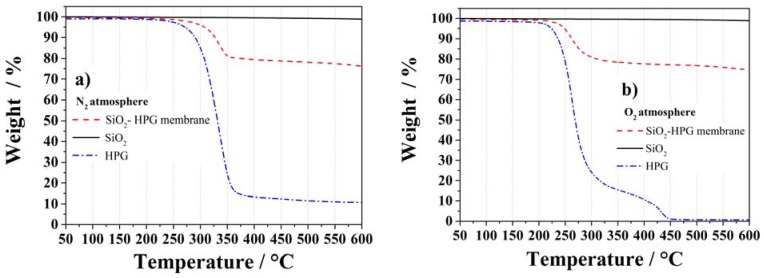
TGA measurements of HPG, SiO_2_ particles and the SiO_2_–HPG membrane in nitrogen (**a**) and oxygen (**b**) atmospheres.

The thermal properties of HPG, SiO_2_ and the complete membrane were investigated in N_2_ and O_2_ atmospheres up to 600 °C via Thermal Gravimetric Analysis (Figure 4). The membrane and its components are thermally stable up to, at least, 200 °C. The less stable component (HPG), in fact, showed a thermal stability up to about 240 °C under N_2_ (Figure 4a), but only ~200 °C under O_2_ (Figure 4b), in agreement with the thermal stabilities of HPG [26]. The thermal decomposition is attributed to the loss of hydroxyl groups as water molecules and the breakdown of the backbone chain of the guar molecule [27] with the somehow lower thermal stability in O_2_ being due to the oxidation of the hydroxyl groups in this atmosphere. At higher temperatures (*i.e.*, >240 °C in N_2_ and >200 °C in O_2_), both the HPG and the membrane showed sharp mass losses. However, while under nitrogen a small but continuous weight loss is observed due to the incomplete binder decomposition (at 600 °C still 10% of the HPG binder weight is still present), under O_2_ atmosphere all HPG binder was lost at 450 °C due to the reaction of carbon (C) and O_2_ forming carbon dioxide CO_2_ [28]. The loss of hydroxyl groups on the particles surface is the reason for the slight weight loss observed for the SiO_2_ in both atmospheres [29]. In fact, the weight loss of the membrane is lower than 75% in both atmospheres, as expected from its SiO_2_ content.

**Figure 5:**
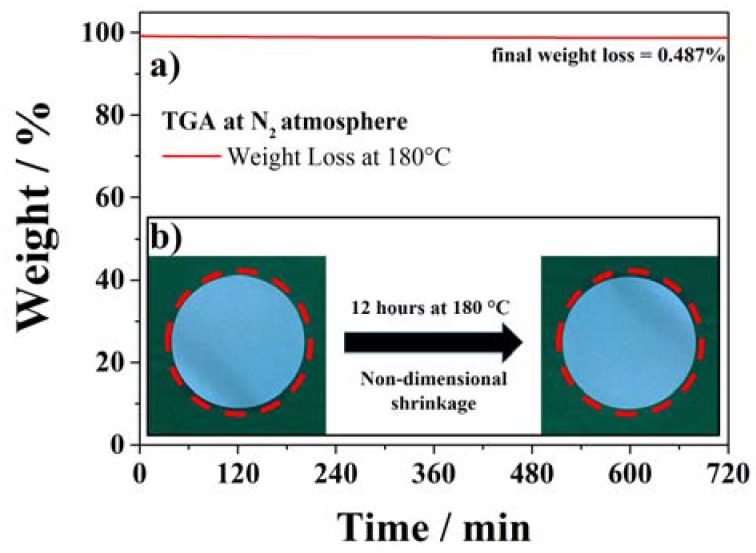
Thermal stability test of SiO_2_–HPG membrane at 180 °C for 12 h (**a**) TGA measurement; (**b**) dimensional-shrinkage test.

Concerning the safety of lithium-ion batteries, the thermo-mechanical stability of the membrane is also very important. The cathode and anode electrodes must be, in fact, physically separated at any time. The shrinkage and/or decomposition of the separator membrane should not take place even at relatively high temperatures, since any direct contact of the two electrodes would induce continuous heat-generation ending in the cell thermal runaway. Therefore, the thermal behavior of the SiO_2_–HPG membrane was evaluated performing a TGA isothermal measurement at 180 °C for 12 h. The results of this test are displayed in Figure 5a. As it can be seen, a negligible weight loss (0.5%) is observed upon the isothermal test at 180 °C for 12 h, which, most likely, originated from the release of remaining moisture in the membrane and the loss of hydroxyl groups from the SiO_2_ particles surface [29]. To verify the thermo-mechanical stability of the membrane, *i.e.*, its dimensional stability upon exposition to high temperatures, a sample was stored at 180 °C for 12 h. Figure 5b compares the size of the same membrane before and after such a storage, showing that no shrinkage or variation in size took place upon the test. Both results indicate that the SiO_2_–HPG membrane can very well stand the exposition to high temperatures without any thermal or mechanical degradation. Additionally, they indicate as the membrane can be handled at high temperatures during the drying process of lithium-ion batteries, allowing for the all-in-one drying step of the electrodes and separator assembly.

### 2.2. Electrochemical Tests

**Figure 6:**
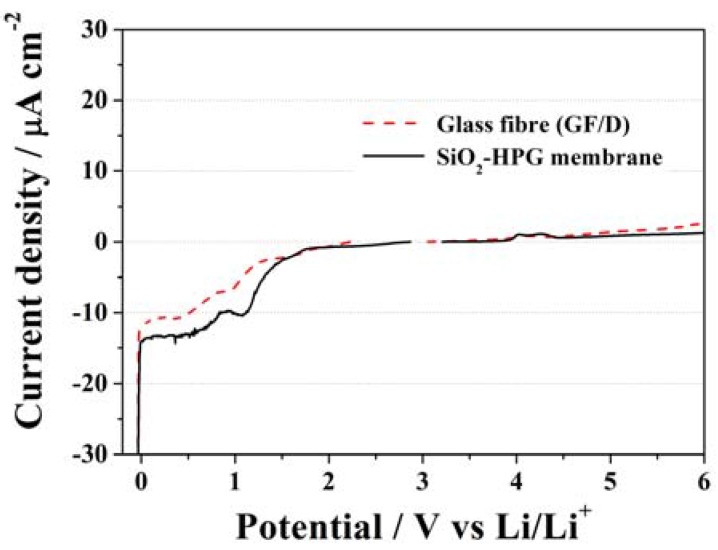
Linear sweep voltammograms of the SiO_2_–HPG membrane and glass fiber separator using 1 mol·L^−1^ (LiPF_6_) dissolved in EC:DMC (1:1) *w*/*w* as electrolyte. Scan rate 0.1 mV·s^−1^.

The electrochemical stability of the SiO_2_–HPG membrane was examined by linear sweep voltammetry (LSV) at a scan rate of 0.1 mV·s^−1^. The LSV results are compared in Figure 6 with those of the glass fiber separator commonly used in laboratory-made lithium-ion cells. The two separators showed a rather similar electrochemical behavior offering wide electrochemical stability windows (ESWs) ranging from the onset of lithium plating at 0 V *vs.* Li/Li^+^ to above 5.0 V *vs.* Li/Li^+^. During the anodic sweep, the SiO_2_–HPG membrane shows no current flow up to, at least, 4.0 V *vs.* Li/Li^+^. Above this potential small features are noticeable, however, these currents are never exceeding the magnitude of 5 µA·cm^−2^ and are always lower than those observed for the commercial glass fiber separator. In the cathodic sweep, the behavior until 1.5 V of both separators is, also, fully comparable. However, for the peak occurring at 1.2 V *vs.* Li/Li^+^ (associated to SEI formation on the Li metal electrode), the current response of the SiO_2_–HPG membrane is higher. The reason of such a behavior might be attributed to the higher reactivity of the hydroxyl groups on guar gum, which can react forming a passive layer on metallic lithium or the higher surface area of the SiO_2_ particle with respect to the glass fiber [30]. Nevertheless, both curves follow the same behavior until 0 V (*vs.* Li/Li^+^), where lithium plating took place. Hence, the SiO_2_–HPG membrane show suitable electrochemical stability for use as a separator in high voltage lithium-ion batteries [10,31].

**Figure 7:**
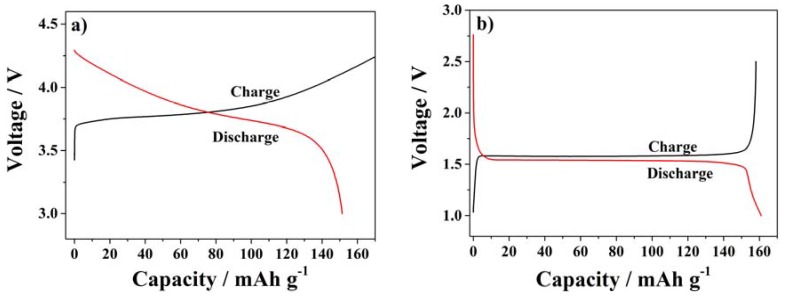
First charge and discharge profile at 0.1C using SiO_2_–HPG membrane as separator in (**a**) NMC; (**b**) and LTO half-cells.

Finally, the SiO_2_–HPG membranes were tested as separators for lithium batteries. Figure 7 shows the first charge and discharge profiles of half-cells comprising NMC or LTO as active materials at 0.1C and 20 °C. Figure 7a depicts the typical sloping profile of NMC *vs.* metallic lithium. At 0.1C, the high discharge capacity of 151 mAh·g^−1^ was delivered by NMC indicating that the separator allows the full redox reaction of the cathode material. The slight irreversibility (the charge capacity is higher than the discharge capacity) is always observed with NMC composite electrodes coated in aqueous binders [32,33,34]. Additionally, Figure 7b shows the typical flat potential profile of Li^+^ (de)insertion of in the LTO spinel structure, associated with the specific discharge capacity of 161 mAh·g^−1^ (at 0.1C). The first cycle potential profiles indicate as the LTO composite electrode [16] performed very well with the SiO_2_–HPG separator. With both electrodes no anomalous reactions or decompositions of the separator were observed. Hence, the cells were subjected to long term cycling tests at different C-rates to further validate the separator stability.

**Figure 8:**
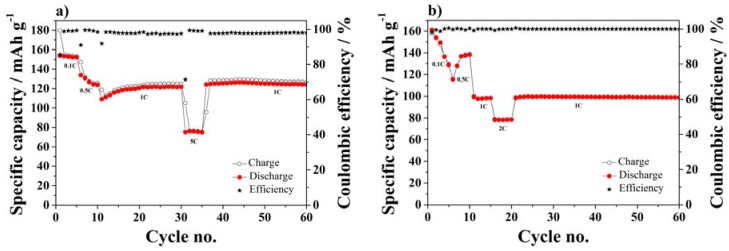
Cycle performance at different C-rates using SiO_2_–HPG membrane as separator in (**a**) NMC; (**b**) and LTO half-cells.

Figure 8 displays the cycle performance in terms of specific capacity *vs.* the number of cycles at different rates of half-cells using NMC (Figure 8a) or LTO (Figure 8b) composite electrodes.

The NMC half-cells show a stable cycle performance during the first 5 cycles at 0.1C, with specific capacities high than 150 mAh·g^−1^. Upon C-rate increase to 0.5C, the capacity decreased, however, increasing again upon further cycling at 1C rate, reaching good performance after 25 cycles. Such a capacity fluctuation is related to the inhomogeneous wetting of the electrode by the electrolyte solution, which, however, takes place within a few further cycles. Moreover, the NMC half-cell also shows good performance at high current density (5C). Finally, very stable performance is observed in the additional cycles at 1C combined with extremely high coulombic efficiencies, especially considering the presence of lithium metal anode in the cell.

Figure 8b shows the cycle performance of the LTO half-cell. Once more, a substantial capacity fading is observed during the initial cycles, which can be, once more, attributed to an incomplete electrode wetting. Nevertheless, after the initial cycles, the cell showed good performance at 0.5C rate with specific capacity of ~140 mAh·g^−1^. Moreover, at high current density of 1C and 2C, the cell show a notable performance stability, where specific capacity of ~100 mAh·g^−1^ and 80 mAh·g^−1^ were reached at 1C and 2C with high coulombic efficiency.

Summarizing, the stable performance evidenced by the long-term cycle tests of half-cells, containing the NMC or LTO composite electrodes, supports for the viable use of the SiO_2_–HPG separator in full lithium-ion cells.

## 3. Experimental Section

### 3.1. Membrane Preparation

Firstly, a binder solution was obtained by the hydration of a predetermined amount of hydroxypropyl guar gum (HPG, Lamberti SpA, viscosity: 3000–5000 cps and pH: 5–7 (1% solution)) in deionized water by magnetic stirring. Separately, SiO_2_ particles (average particle size d_50_ ~1 µm, Shott AG) were dispersed in deionized water by vigorous stirring and ultrasonication. This process was applied to destroy agglomerates of ceramic particles, which would affect the morphology of the final membrane. The binder solution and the SiO_2_ dispersion were mixed by magnetic stirring until homogeneous slurries were obtained.

These were cast on a Polytetrafluorethylene (PTFE) tape, using a glass bar, and then dried at room temperature (20 °C) and subsequently at 80 °C in an atmospheric oven. After drying, the membranes were removed from the PTFE tape. Before testing, the prepared membranes were dried under vacuum at 120 °C for at least 12 h to remove remaining moisture.

### 3.2. Membrane Characterization

The surface and the cross-sectional morphologies of the membranes were investigated using a ZEISS 1550VP Field Emission SEM (Carl Zeiss). The porosity P (%) of the membrane was estimated by liquid absorption test using hexadecane (ReagentPlus^®^) [5]. The porosity measurements were performed with eight different samples from each membrane. In particular, a piece of the membrane was weighed before and after dipping in hexadecane for 30 min. The excess hexadecane on the surface was wiped using filter paper. The porosity was calculated using Equation (1).

(1) $$P=\frac{w_{T}-w_{S}}{d_{H}\times V_{S}}\;\times 100$$

where *w_S_* is the weight of the pristine separator, *w_T_* is the weight of separator with the absorbed hexadecane, *d_H_* is the hexadecane density (0.773 g/mL) and *V_S_* is the volume of the separator [9].

The thermal properties of the membranes were evaluated by Thermogravimetric Analysis (TGA) and dimensional-shrinkage measurement. The TGA experiments of pure HPG and SiO_2_, and the membranes were carried out by using a Q 5000 IR TGA instrument (TA Instruments) with a temperature ramp of 10 °C·min^−1^ up to 600 °C in both N_2_ and O_2_ atmospheres. The dimensional shrinkage was evaluated determining the membrane’s areal change after 12 h storage at 180 °C under N_2_ [10,11].

The separator’s affinity towards the liquid electrolyte (*i.e.*, its uptake) is an important factor influencing the performance of batteries [5,7,8,9]. For investigation of the wettability and the electrolyte uptake ability for the SiO_2_–HPG membranes, the commercial electrolyte consisting of 1 mol·L^−1^ solution of lithium hexafluorophosphate (LiPF_6_) in a mixture of ethylene carbonate and dimethyl carbonate EC:DMC (1:1 *w*/*w*) (BASF, LP 30) was used. For the electrolyte uptake, a piece of membrane was weighted before and after soaking into the electrolyte for 1 h. These experimental measurements were carried out in a dry-room (R.H. < 0.1%), five times with different samples. The electrolyte uptake U (%) was calculated by using Equation (2).

(2) $$U=\frac{w_{1}-w_{0}}{w_{0}}\;\times 100$$

where *w*_0_ is the weight of the dry separator and *w*_1_ is the weight of the separator after absorbing the electrolyte [11]. A visual analysis of the membrane wetting speed was performed by comparing the pictures of the pristine membrane before and after a small amount of electrolyte (80 µL) was dropped on the membrane [5,7,10].

### 3.3. Electrochemical Tests

Three-electrode cells (Swagelok cell) were used to evaluate the electrochemical compatibility of SiO_2_–HPG membrane as separator for lithium-ion batteries. The cells were assembled in an argon-filled glove box using metallic lithium (ROCKWOOD LITHIUM, battery grade) as counter and reference electrodes. A disc of the membrane was placed between the electrodes and soaked with the electrolyte (LP30), while a glass fiber separator (WHATMAN, GF/D) was used as separator for the reference electrode. The electrochemical stability window (ESW) of the membranes was tested using aluminum and nickel discs as working electrode for, respectively, the anodic and cathodic sweeps. The measurements were performed using a VMP3 (BioLogic, Grenoble, France). A scan rate of 0.1 mV·s^−1^ was applied from the open circuit potential to 6 V and −2 V, respectively, for the anodic and cathodic sweeps.

NMC and LTO electrodes were prepared to test the stability and performance of the SiO_2_–HPG membranes as separators for lithium-ion batteries. As active cathode material, commercially available NMC (TODA, average particle size d_90_ = 10 µm) was used, while LTO (Süd Chemie AG, Munich, Germany) was used as anode active material. Both electrodes were prepared using sodium carboxymethyl cellulose (Na-CMC, DOW WOLFF CELLULOSICS, Walocel CRT 2000 PPA 12) with a degree of substitution of 1.2 as binder, while the conducting carbon black was C-NERGY Super C45 (IMERYS, primary average particle size: 30 nm). Both electrodes were prepared in the same way. First, the CMC binder was dissolved in deionized water using magnetic stirring and subsequently a predetermined amount of Super C45 was added. After 3 h of continuous mixing, the active material was added and the electrode slurries were stirred for 1 h. Further dispersion at medium stirring speed (5000 rpm) was performed with a high-speed mixer (Dremel 4000-4/65). For preventing aluminum current collector corrosion, the pH was controlled as described by Kim *et al.* [35]. The final homogenous slurry was cast on aluminum foil (thickness: 20 µm) using a laboratory scale doctor blade coater. The coated aluminum foils were pre-dried in an atmospheric oven at 80 °C and at 180 °C under vacuum for 12 h prior performing electrochemical investigations. NMC and LTO electrodes were prepared with the same material composition: 88 wt % active material, 7 wt % Super C45, and 5 wt % CMC. The electrodes were pressed at 10 kg·cm^−2^ using a manual press for 1 min to reduce the electrodes’ thickness and porosity, enhance the adherence of the active material layers to the substrate, and make the electrodes’ surface uniform.

Half-cells (Li metal anode) were prepared using NMC and LTO disc electrodes with an area of 1.13 cm^−2^. The cells were assembled in a pouch bag configuration using aluminum tabs for the NMC and LTO electrodes, while nickel tabs were used for the lithium electrode. The SiO_2_–HPG membrane was used as separator. It was placed between the electrodes and soaked with the electrolyte. The cells were assembled in a dry-room (R.H. < 0.1%) at 20 °C ± 1 °C and tested using a MACCOR Battery tester 4300 (Tulsa, OK, USA) inside climatic chambers (Binder KB 400, 20 °C ± 0.1 °C). The galvanostatic charge/discharge tests of NMC half-cells were carried out between 3.0 V and 4.3 V *vs.* Li/Li^+^, while those of LTO electrodes were performed between 1.0 V and 2.5 V *vs.* Li/Li. The galvanostatic tests were performed at different C-rates (0.1C, 0.2C, 1C, 2C, and 5C).

## 4. Conclusions

In this work, an environmentally friendly process to prepare a high temperature stable separator for lithium-ion batteries was introduced. The self-standing separator was prepared using low cost materials, such as silicon dioxide and hydroxypropyl guar gum, through aqueous processing. The physical characteristics of the separator made with the new process, such as porosity of 52% and the homogeneous distribution of pores, are suitable for lithium-ion battery applications. The SEM morphological analysis showed pores smaller than 1 µm on both surfaces, however, larger pores (>1 µm) are observed in the cross-sectional images, which can function as an electrolyte reservoir, in addition to allowing Li^+^ ion transport during the charge and discharge. The separator membrane shows exceptionally good thermal stability at high temperatures without any significant dimensional-shrinkage. After applying 180 °C for 12 h a weight loss smaller than 0.50%, with no relevant decomposition, was observed at ~240 °C in N_2_ and at ~200 °C in O_2_. Linear sweep voltammetry showed as the SiO_2_–HPG membrane separator is electrochemically stable in a wide potential range. More important, it demonstrates good performance in half-cells (NMC/metallic lithium and LTO/metallic lithium) with remarkable cycle stability, despite a slightly more marked capacity fading on the first cycle for both materials. Further studies are ongoing to clarify and solve this issue, allowing the use of SiO_2_–HPG membrane separator in full lithium-ion cells.
